# Exploring ethical elements in reporting guidelines: results from a research-on-research study

**DOI:** 10.1186/s41073-025-00180-0

**Published:** 2025-09-22

**Authors:** Clovis Mariano Faggion, Carla Brigitte Susan Kohl

**Affiliations:** Department of Periodontology and Operative Dentistry, University Hospital Münster, Waldeyerstraße 30, 48149 Münster, Germany

**Keywords:** Conflict of interest, Ethics, Ethics in publishing, Reporting guidelines, Reporting checklists, Transparency, EQUATOR

## Abstract

**Background:**

Reporting guidelines are key tools for enhancing the transparency and reproducibility of research. To support responsible reporting, such guidelines should also address ethical considerations. However, the extent to which these elements are integrated into reporting checklists remains unclear. This study aimed to evaluate how ethical elements are incorporated in these guidelines.

**Methods:**

We identified reporting guidelines indexed on the “Enhancing the Quality and Transparency of Health Research (EQUATOR) Network” website. On 30 January 2025, a random sample of 128 reporting guidelines and extensions was drawn from a total of 657. For each, we retrieved the associated development publication and extracted data into a standardised table. The assessed ethical elements included COI disclosure, sponsorship, authorship criteria, data sharing guidance, and protocol development and study registration. Data extraction for the first 13 guidelines was conducted independently and in duplicate. After achieving 100% agreement, the remaining data were extracted by one author, following “A MeaSurement Tool to Assess Systematic Reviews” (AMSTAR)-2 recommendations.

**Results:**

The dataset comprised 101 original guidelines and 27 extensions of existing guidelines. Half of the included guidelines were published from 2015 onward, with 32.0% published between 2020 and 2024. The median year of publication was 2016. Approximately 90 of the 128 assessed guidelines focused on clinical studies.

Over 70% of the guidelines did not include items related to conflicts of interest (COI) or sponsorship. Only 8.6% addressed COI and sponsorship jointly in a single item, while fewer than 9% covered them as two separate items. Notably, only two guidelines (1.6%) provided instructions for using the ICMJE disclosure form to report potential conflicts of interest.

Nearly 20% of the guidelines offered guidance on study registration. Fewer than 30% recommended the development of a research protocol, and only 18.8% provided guidance on protocol sharing. Additionally, fewer than 10% of the checklists included guidance on authorship criteria or data sharing.

**Conclusion:**

Ethical considerations are insufficiently addressed in current reporting guidelines. The absence of standardised items on COIs, funding, authorship, and data sharing represents a missed opportunity to promote transparency and research integrity. Future updates to reporting guidelines should systematically incorporate these elements.

**Supplementary Information:**

The online version contains supplementary material available at 10.1186/s41073-025-00180-0.

## Introduction

Reporting guidelines are essential tools that assist authors in clearly and comprehensively communicating their research. When properly applied, these documents ensure that readers receive detailed information regarding a study’s methodology and findings, enabling accurate interpretation and identification of potential biases. Inadequate reporting, in contrast, can significantly hinder interpretation, as it obscures potential sources of bias [1] that may have influenced the results.

Among the most widely used guidelines are those developed by the Enhancing the Quality and Transparency of Health Research (EQUATOR) Network [2].

The EQUATOR Network, established in 2006, serves as a central repository for reporting guidelines in health research, with the overarching aim of enhancing the reliability and transparency of research reporting. It maintains a continually updated online library of resources and actively promotes the use of reporting guidelines through education, training, and institutional support. Furthermore, the Network supports the development, dissemination, and implementation of robust guidelines, conducts research to enhance the quality of health research, and coordinates a global network of local centres to strengthen research reporting practices worldwide [2].

Authors submitting manuscripts to medical journals, including this one, are often required to adhere to these guidelines and include them as part of their submissions. For instance, the use of the CONSORT guideline is recommended for the conduct of randomised trials [3]; the STROBE recommendations serve as guidelines for observational studies [4]; and the PRISMA guidance can be used when performing systematic reviews and meta-analyses [5]. These guidelines may be useful for peer reviewers [6], as they offer a standardised framework for assessing the completeness and ethical transparency of submitted manuscripts. In particular, clear instructions regarding the disclosure of conflicts of interest (COI) and sponsorship information may enable reviewers to identify potential biases and contribute to a more objective and reliable evaluation process.

Another potential use of these guidelines is their application by clinicians as a tool for learning research methodology [7]. Clinicians can use the individual guideline items as foundational points for further exploration and study of the methodological topics they address.

Reporting guidelines are designed to serve as templates for the conduct and reporting of methodology, results, and discussion, e.g. encompassing study design, baseline participant data, intervention details, outcome definitions, and statistical analyses. This ensures the reader's ability to understand and interpret the study, including its significance, providing transparency and facilitating the replication of results. Guidelines aim to promote research integrity across a range of dimensions, not solely limited to ethical disclosures. However, one essential domain among several areas addressed by reporting guidelines is ethical considerations, including the disclosure of conflicts of interest and funding sources, the sharing of data, guidance on authorship, and the establishment and sharing of protocols.

Normative standards for ethical elements such as declarations of COIs and funding or sponsorship are addressed in broader ethical frameworks, including the Declaration of Helsinki [8] and the Council for International Organizations of Medical Sciences (CIOMS) guidelines [9]. However, these frameworks are not intended as operational tools for manuscript preparation or peer review. In contrast, reporting guidelines are specifically designed as practical tools, offering structured checklists to facilitate transparent and comprehensive reporting. Moreover, recent evidence [10] highlights persistent underreporting of COI and sponsorship information across key sources, including journal websites, full-text articles, and indexing databases. This suggests that relying solely on general ethical codes may be insufficient to ensure consistent transparency in practice.

Recent studies [11, 12] indicate that many researchers lack a clear understanding of key ethical components—such as authorship criteria, data sharing practices, and trial registration—or face barriers to their implementation. These challenges are often associated with insufficient ethics training, inconsistent oversight, and uncertainty regarding institutional or international requirements. Integrating ethical elements into reporting guidelines could help mitigate these issues by reinforcing ethical standards during the reporting phase. This strategy would promote consistency across publications and support peer reviewers in assessing manuscripts against clear, standardised criteria for ethical transparency.

Despite the importance of ethical transparency, there is limited literature examining the extent to which EQUATOR guidelines incorporate ethical considerations within their checklist items. This gap in understanding is particularly relevant for researchers and guideline developers, as it may highlight an area in need of more thorough integration.

Several suggestions have been proposed for a methodological framework to guide the development of reporting guidelines, with the aim of enhancing transparency and reproducibility across various research disciplines [13].

Accordingly, this study aims to evaluate the extent to which reporting guidelines address ethical elements in their items, thereby contributing to the broader conversation on research transparency and integrity.

## Methods

### Research question

A research question was developed to guide this study: “To what extent do existing reporting guidelines incorporate ethical elements such as conflict of interest, authorship criteria, data sharing, and trial registration?”.

### Eligibility Criteria

Only reporting guidelines available on the EQUATOR Network website were included in this study. The EQUATOR Network serves as a comprehensive repository of resources designed to enhance the reporting of pre-clinical, clinical, and secondary research (e.g., systematic reviews). These guidelines are widely recognised and frequently used within the research community. Reporting guidelines not listed on the EQUATOR website were excluded from the analysis.

### Extensions of guidelines

Extensions of existing guidelines were included and classified as either “official” or “unofficial.” According to a statement on the CONSORT and SPIRIT websites [14], extensions are not permitted to use the SPIRIT or CONSORT name unless they are developed in collaboration with the original guideline development group. In some instances, studies proposed extensions to existing reporting guidelines (e.g., CONSORT or SPIRIT) without such collaboration. These were therefore classified as “unofficial extensions.”

The same classification criteria were applied to extensions derived from other established guidelines: if an extension was not developed in collaboration with, or by, the original guideline group, it was categorised as “unofficial.”

Additionally, when accessing “official” guidelines through the EQUATOR Network, we observed references to broader or more general guidelines produced by the same group. These were likewise classified as “official.”

### Search and selection of reporting guidelines

Reporting guidelines were identified through a systematic search of the EQUATOR Network website [15]. As of 22 January 2025, the website listed 657 reporting guidelines. To determine an appropriate sample size, a pilot study was conducted using 25 guidelines (see Supplementary File: Tables 1 and 2), with the presence or absence of a COI item as the outcome measure. The EQUATOR website was initially assessed for this purpose on 10 October 2024.

In this preliminary analysis, 36% of the guidelines included such an item. Based on this proportion and a margin of error of 8%, the required sample size was calculated to be 115 guidelines.

On January 30, 2025, a random sample of reporting guidelines was selected using the online tool Research Randomizer (https://www.randomizer.org/). No restrictions were imposed on the time of publication of the guidelines or the study type. A final sample of 128 reporting checklists was drawn from the 657 available on the EQUATOR website. While a comprehensive assessment of all available guidelines would have been ideal, it was not feasible due to resource constraints. However, a robust sampling strategy was employed to ensure representativeness within practical limits. Given the continuous growth of reporting guidelines on the EQUATOR Network—from 657 in January to 672 by 18 July 2025—a slightly larger random sample was selected to enhance the accuracy of our findings.

### Data extraction

Before data extraction, an additional training session was conducted to ensure reproducibility and calibrate the authors’ understanding of the extraction items. Thirteen checklists were independently assessed by both authors, achieving perfect agreement (100%). Based on this high level of consistency, the remaining checklists were assessed by a single author [16]. The full text of each reporting guideline was systematically examined for ethical content, with particular attention to COI and sponsorship. Each reporting guideline was assessed using predefined criteria (items a–p), with information recorded through a combination of binary responses (e.g., yes/no), ordinal scales (e.g., a 1–6 rating for the presentation of COI and sponsorship), and brief descriptive comments when applicable. The full description of the variables extracted is reported in the supplementary file.

- the name of the guideline.
- year of publication.
- type of guideline (main or extension).
- the name of the guideline that is extended.
- type of extension.
- type of study addressed by the guideline.
- item description and rationale.
- whether the guideline explicitly recommends the use of the International Committee of Medical Journal Editors (ICMJE) disclosure of interest form.
- whether the guideline includes authorship guidance.
- whether it provides recommendations for sharing raw data related to the study.
- presentation of COI and sponsorship.
- the content of the item related to COI guidance.
- the content of the item related to sponsorship guidance.
- whether guidance is provided for study registration.
- whether the guideline recommends establishing a research protocol prior to conducting the study.
- whether it encourages authors to share the research protocol.

### Data analysis

All data were descriptively statistically analysed using Microsoft Excel; continuous variables are reported as medians with interquartile ranges (IQR).

## Results

### Characteristics of Reporting Guidelines

A total of 128 documents were analysed, comprising 101 reporting checklists and 27 checklist extensions. The median year of publication was 2016 (IQR: 2011–2021), and the number of publications increased steadily over five-year intervals. The highest number of documents was published in 2021 (12/128; 9.4%), with the most active publication period occurring between 2020 and 2024.

Among the 27 extensions, the majority were classified as official extensions (17/128; 13.3%). Specifically, 14 were extensions to CONSORT (14/128; 10.9%), five to STROBE (5/128; 3.9%), two each to STARD and RIGHT (2/128; 1.6%), and one extension each to PRISMA, STROBE (in conjunction with RECORD), SQUIRE, and CARE (1/128; 0.8%).

The sample represented a diverse range of reporting guideline types. More than two-thirds of the documents (90/128; 70.3%) were related to clinical research, including observational studies, randomised controlled trials (RCTs), and experimental studies. Approximately 10% (12/128; 9.4%) focused on secondary research, such as systematic reviews, meta-analyses, and guideline development. Of these, two also addressed secondary research in the context of animal studies.

Regarding the presentation of checklist items, 43 documents (33.6%) provided both a description and a rationale or examples for each item. In 68 cases (53.1%), a description was provided without accompanying rationale or examples. Seventeen documents (13.3%) included neither detailed descriptions nor justifications for the items.

Comprehensive characteristics of the reporting guidelines are presented in Table 1.

**Table 1:** Characteristic of the reporting guidelines/extensions (*N* = 128)

**Characteristic**		*N*** (%)**
Years of publication of the guidelines/extensions	1995–1999	3 (2.3)
	2000–2004	4 (3.1)
	2005–2009	13 (10.2)
	2010–2014	31 (24.2)
	2015–2019	36 (28.1)
	2020–2024	41 (32.0)
Type of records	Reporting checklist	101 (78.9)
	Extension to guideline	27 (21.1)
Type of extension	Official	17 (13.3)
	Non-official	9 (7.0)
	Unclear	1 (0.8)
Extension to the following guideline^a^:	CONSORT	14 (10.9)
	STROBE	6 (4.7)
	RIGHT; STARD	2 (1.6)
Study types^a^	Clinical studies	90 (70.3)
	Other	23 (18.0)
	Secondary research	12 (9.4)

^a^ The three most prevalent results are shown

### Ethical Elements in Reporting Guidelines

Seventeen reporting guidelines or extensions included an item solely addressing sponsorship or funding (17/128; 13.3%), without any mention of COI. A total of 21 documents referenced COI (21/128; 16.4%). Of these, ten included COI and sponsorship/funding information in a single item (10/128; 7.8%), while eleven included separate items addressing each topic (11/128; 8.6%). Notably, none of the documents included an item that addressed COI exclusively.

Overall, the majority of documents (90/128; 70.3%) did not include any item related to either COI or sponsorship/funding.

Only two studies (2/128; 1.6%) recommended the use of the ICMJE disclosure form or encouraged authors to disclose all potential COIs, including those that may not be recognised by the authors themselves.

Furthermore, most guidelines and extensions did not recommend study registration (103/128; 80.5%), nor did they encourage the establishment (94/128; 73.4%) or sharing (104/128; 81.2%) of study protocols.

Detailed information on the ethical components of the reporting guidelines is provided in Table 2.

**Table 2:** Ethical elements in reporting guidelines (*N* = 128)

**Ethical element**		*N*** (%)**
Presentation of the guidance on conflicts of interest and funding disclosure	Conflict of interest (COI) item presented exclusively	0 (0.0)
	Sponsorship/funding item presented exclusively	17 (13.3)
	COI and sponsorship/funding addressed jointly within a single item	10 (7.8)
	COI and sponsorship/funding addressed in two distinct items	11 (8.6)
	Neither COI nor sponsorship/funding item is presented^a^	90 (70.3)
Explicit disclosure of conflicts of interest provided?	Yes	2 (1.6)
	No	126 (98.4)
Instructions to use the ICMJE disclosure of interest form provided?	Yes	2 (1.6)
	No	126 (98.4)
Guidance on authorship (positions) provided?	Yes	7 (5.5)
	No	121 (94.5)
Guidance on sharing raw data provided?	Yes	11 (8.6)
	No	117 (91.4)
Guidance on registration of the study provided?	Yes	25 (19.5)
	No	103 (80.5)
Guidance on research protocol establishment provided?	Yes	34 (26.6)
	No	94 (73.4)
Guidance on sharing the research protocol provided?	Yes	24 (18.8)
	No	104 (81.2)^b^

^a^ One guideline (1/128; 0.8%) neither had a dedicated item for COI or sponsorship/funding, but relevant information was mentioned elsewhere in the text

^b^ The largest remainder method was used for rounding

## Discussion

### Main Findings

This study examined how ethical elements are addressed in reporting guidelines indexed on the EQUATOR website. The findings from 128 reporting guidelines indicate that ethical considerations are inadequately addressed.

### Interpretation of Findings

Half of the reporting guidelines and extensions in the present sample were published from 2015 onwards. This might suggest that ethical aspects of research—such as clear reporting and standards of reporting to allow reproducibility of findings [17]—are receiving more attention from the scientific community. However, although more attention seems to have been paid to ethical aspects of research, reporting guidelines still have several limitations regarding how these elements are described.

### COI and sponsorship disclosure

One important ethical element that should be well described in a reporting guideline is how authors should report their own COI. Impressively, the great majority of the guidelines/extensions did not include any item for authors to report information about potential COIs. Furthermore, a portion of the reporting guidelines addressed COI and sponsorship in a single item. This combined reporting may confuse authors regarding the distinction between COI and sponsorship. Sponsorship is sometimes seen as a synonym for COI; however, the concept of COI is broader and may involve financial aspects (in the form of sponsorship, for example) and non-financial aspects [18]. We believe that including two distinct items for financial and non-financial COI would clarify these concepts for authors.

Discrepancies in the disclosure of COI statements have been observed in both publications and conference settings [19–21]. For instance, in medical conferences, COI statements in oral presentations were frequently absent, unclear, or inconsistent with the corresponding written versions. Such inconsistencies may hinder the audience’s ability to assess the objectivity and quality of the information presented [21].

Regarding the reasons for inaccurate COI reporting, it is plausible that such discrepancies may occur either intentionally—as a form of misconduct—or unintentionally, due to misinterpretation of relevant policies or guidelines. One study, for example, found that most attendees at orthopaedic society meetings misinterpreted the disclosure requirements [20].

These findings suggest that, even when reporting guidelines include items related to COI or funding disclosures, misinterpretation may still occur. Therefore, it is essential that these items are standardised, clearly worded, and not open to subjective interpretation. A critical strategy for ensuring the reliability of COI disclosures is to require authors to declare all relationships with third parties, rather than relying on their own judgment of what constitutes a potential conflict of interest. This approach shifts the responsibility for interpretation to the reader, rather than the author [22].

The International Committee of Medical Journal Editors (ICMJE) provides standardised forms for disclosing COI; the use of these forms can promote uniformity and reduce the risk of misinterpretation. Concerning the reporting of potential COI by authors using the guidelines, only two guidelines recommended using the ICMJE form for declaring COI. It would be important for reporting guidelines to align with the expectations of most scientific journals, which often require authors to submit such a form when submitting manuscripts.

Likewise, reporting guidelines should clearly state that authors are required to disclose sponsorship or funding sources (separately) from the declaration of the COI. This promotes transparency and enables editors, peer reviewers, and readers—especially clinicians who apply findings in patient care—to assess potential biases in the reported results. It has been shown that industry-sponsored studies are significantly more likely to report favourable outcomes [23], independent of methodological quality [24] and that drug and device studies funded by manufacturers tend to show more positive efficacy results than those without sponsorship, referred to as “industry bias” [25]. Thus, standardised disclosures towards sponsorship and funding would promote transparency and reduce the risk of misinterpretation of the expressiveness of publications.

### Authorship criteria

Another important ethical element that could be better addressed in reporting guidelines is the provision of guidance on authorship roles. Nearly 95% of the reporting guidelines and extensions analysed did not include any item concerning authorship recommendations. Including such guidance would be pivotal in mitigating instances of gift and ghost authorship [26]. Authorship abuse can occur when individuals, such as department heads, exploit their authority to be listed as co-authors on publications despite having made no meaningful contribution to any stage of the research. Including clear recommendations on how authorship should be reported would help curb such unethical practices, particularly in cases where early-career researchers are vulnerable to pressure or coercion by more senior academics.

### Data sharing guidance

More than 90% of the included reporting guidelines/extensions did not report any item regarding data sharing. Sharing research data (called here the “raw data”) is a pivotal component for raising ethical standards in research and enabling the reproducibility of findings [27]. Furthermore, by sharing data, other research teams can use it to develop new projects and hypotheses that could accelerate scientific progress [28]. However, data sharing also entails risks for certain types of research, such as clinical studies. For example, it may lead to privacy invasions or breaches of confidentiality for patients included in a trial [28]. Patients may also be put at risk if the data is incorrectly analysed or interpreted by secondary research teams. In the case of secondary research, such as systematic reviews or meta-research, there is no reason not to share the data used in these studies, since the data (from systematic reviews or meta-research) is already fully available in the public domain, although in some specific cases the data were obtained from the authors of primary studies included in the review. A recent position paper from the EQUATOR Network advocates for including data sharing as a checklist item in all reporting guidelines [29].

### Protocol development and study registration

Protocol development and study registration are important steps in research, particularly for identifying potential biases, such as selective outcome reporting [30]. Authors may choose to report only selected outcomes that serve their interests, omitting others due to potential COIs. For instance, consider a randomised controlled trial evaluating a new therapy for cancer, compared to a conventional therapy. If the primary outcome shows no difference in efficacy, authors may instead choose to report a secondary outcome that favours the new therapy. Beyond being unethical, this behaviour could result in patient harm, as clinical decisions may be based on misleading information. Such practices would be less likely if a publicly registered protocol were available, enabling comparison between planned and reported outcomes. Several meta-research studies have already focused on identifying selective outcome reporting by assessing public registries such as ClinicalTrials.gov [31–34]. Therefore, reporting guidelines could include an item providing guidance on the establishment and reporting of a study protocol.

### Current Advancements in the Reporting of Ethical Elements

A significant recent development is the publication of the CONSORT 2025 guideline in April 2025, which introduces key updates aimed at enhancing transparency in clinical trial reporting [3]. For the first time, CONSORT includes a dedicated item on data sharing (Item 4), specifying requirements for the disclosure of individual de-identified participant data, statistical code, and related materials.

Additionally, funding and conflicts of interest are now addressed more explicitly in Item 5, which has been divided into two distinct sections [3, 35], reflecting a stronger commitment to identifying and disclosing potential sources of bias [35]. These sections are also individually assessed and discussed in detail in the accompanying Explanation and Elaboration document, which offers clearer differentiation between conflicts of interest and sources of sponsorship or funding.

This update establishes an important precedent for CONSORT extensions, which are now expected to align with these enhanced transparency standards. These changes may also serve as a catalyst for revisions to related reporting guidelines.

### Limitations and Strengths

This study has some limitations. Although the number of reporting guidelines and extensions analysed is representative, not all guidelines listed in EQUATOR were included. Furthermore, while EQUATOR guidelines are likely among the most widely used, other reporting guidelines may exist. The major strength of this study lies in its novelty—it is the first to systematically assess and identify methodological limitations concerning ethical elements in reporting guidelines.

### Further Developments

Ethical considerations should be more systematically integrated into reporting guidelines. The scientific community, in particular, ought to engage in a comprehensive dialogue regarding the inclusion of declarative elements—such as COI disclosures, Institutional Review Board (IRB) approvals, funding sources, data and code sharing practices, Open Researcher and Contributor IDs (ORCIDs), return of results (ROR) procedures, and authorship and affiliation guidelines—where applicable, across all reporting standards. Moreover, to promote greater replicability, reporting guidelines should also address additional ethically pertinent aspects, including the evaluation of readability scores, detection of plagiarism, and the adoption of clear and appropriate reporting formats, such as the use of accurate graphical representations, mathematical expressions, and rigorous statistical reporting.

From a broader perspective, updated reporting guidelines concerning ethical elements should be integrated with wider efforts to enhance transparency and research integrity, particularly in relation to responsible research practices and open science [36]. Moreover, the revision of these guidelines should involve diverse stakeholders—including clinicians, statisticians, methodologists, patients, and ethicists—to ensure that ethical considerations are comprehensively addressed.

## Conclusions

Reporting guidelines exhibit methodological limitations regarding the guidance provided to authors on ethical elements related to their research. The findings of this study may support future improvements by helping developers of reporting guidelines to address these ethical issues in upcoming versions.

## Supplementary Information

Supplementary Material 1.

Supplementary Material 2.

## Data Availability

All data generated or analysed during this study are included in this published article [and its supplementary information files].

## Abbreviations

IQR: Interquartile range
COI: Conflict of interest
IRB: Institutional Review Board
ORCID: Open Researcher and Contributor ID
ROR: Return of results
