# Supplementary material for: Exploring ethical elements in reporting guidelines: results from a research-on-research study
Source: Res Integr Peer Rev. 2025 Sep 22;10:20. doi: 10.1186/s41073-025-00180-0 (PMC12452000; doi:10.1186/s41073-025-00180-0)
Supplement: Supplementary file 1 — Supplementary Material 1. [file 41073_2025_180_MOESM1_ESM.docx]

**Supplementary file**

Table 1. Reporting checklists (N=25) with the COI statements (when COI statements were not available, information on funding was reported)

| **Checklist** | **Year** | **Section** | **COI information** |
| --- | --- | --- | --- |
| STROBE | 2007 | Other information | Funding: item 22: Give the source of funding and the role of the funders for the present study and, if applicable, for the original study on which the present article is based |
| CARE | 2013 | No item about COI | No item about COI |
| STARD | 2015 | Other information | Item 30: Sources of funding and other support; role of funders |
| CHEERS | 2022 | Other relevant information | Conflicts of interest: item 28: Report authors conflicts of interest according to journal or International Committee of Medical Journal Editors requirements |
| STREGA | 2009 | Other information | Funding: item 22: Give the source of funding and the role of the funders for the present study and, if applicable, for the original study on which the present article is based |
| MOOSE | 2000 | Reporting of conclusions should include | Item 35: Disclosure of funding source |
| STROBE-nut | 2017 | Other information | Funding: item 22: Give the source of funding and the role of the funders for the present study and, if applicable, for the original study on which the present article is based |
| TRIPOD | 2015 | Funding | Item 22: Give the source of funding and the role of the funders for the present study |
| TRIPOD-SRMA | 2023 | Other information | Competing interests: item 25: Declare any competing interests of review authors |
| TRIPOD+AI | 2024 | Open science | Conflicts of interest: item 18b: D;E: Declare any conflicts of interest and financial disclosures for all authors |
| SPIRIT | 2013 | Ethics and dissemination | Declaration of interests: item 28: Financial and other competing interests for principal investigators for the overall trial and each study site |
| PRISMA | 2020 | Other information | Competing interests: item 26: Declare any competing interests of review authors. |
| SRQR | 2014 | Other | Conflicts of interest: item S20: Potential sources of influence or perceived influence on study conduct and conclusions; how these were managed |
| STROBE-MR | 2021 | Other information | Conflicts of interest: item 20: All authors should declare all potential conflicts of interest |
| SQUIRE | 2016 | Ethical considerations | Item 12: Ethical aspects of implementing and studying the intervention(s) and how they were addressed, including, but not limited to, formal ethics review and potential conflict(s) of interest |
| CONSORT | 2010 | Other information | Funding: item 25: Sources of funding and other support (such as supply of drugs), role of funders |
| CONSORT-abst | 2008 | Conclusions | Item Funding: Source of funding |
| CONSORT-harms | 2022 | Other information | Funding: item 25: Sources of funding and other support (such as supply of drugs), role of funders |
| CONSORT-non-inferiority | 2012 | Other information | Funding: item 25: Sources of funding and other support (such as supply of drugs), role of funders |
| CONSORT Cluster | 2012 | Other information | Funding: item 25: Sources of funding and other support (such as supply of drugs), role of funders |
| ARRIVE | 2020 | Declaration of interests | Item 21: a. Declare any potential conflicts of interest, including financial and nonfinancial. If none exist, this should be stated.  b. List all funding sources (including grant identifier) and the role of the funder(s) in the design, analysis, and reporting of the study |
| CHERRIES | 2004 | No item about COI | No item about COI |
| COREQ | 2007 | No item about COI | No item about COI |
| PRISMA-P | 2015 | Support | Sources 5a: Indicate sources of financial or other support for the review  Sponsor 5b: Provide name for the review funder and/or sponsor  Role of sponsor or funder 5c: Describe roles of funder(s), sponsor(s), and/or institution(s), if any, in developing the protocol |
| TREND | 2004 | No item about COI | No item about COI |

Table 2. Reporting checklists (N=25) with the respective rationale for guiding authors to report potential COI

| **Checklist** | **Rationale** |
| --- | --- |
| STROBE | Explanation: Some journals require authors to disclose the presence or absence of financial and other conflicts of interest. Several investigations show strong associations between the source of funding and the conclusions of research articles. The conclusions in randomized trials recommended the experimental drug as the drug of choice much more often (odds ratio 5.3) if the trial was funded by for-profit organisations, even after adjustment for the effect size. Other studies document the influence of the tobacco and telecommunication industries on the research they funded. There are also examples of undue influence when the sponsor is governmental or a non-profit organization. Authors or funders may have conflicts of interest that influence any of the following: the design of the study; choice of exposures, outcomes, statistical methods, and selective publication of outcomes and studies. Consequently, the role of the funders should be described in detail: in what part of the study they took direct responsibility (eg, design, data collection, analysis, drafting of the manuscript, decision to publish). Other sources of undue influence include employers (eg, university administrators for academic researchers and government supervisors, especially political appointees, for government researchers), advisory committees, litigants, and special interest groups |
| CARE* | There is no “explanation and elaboration” article |
| STARD | Explanation: Sponsorship of a study by a pharmaceutical company has been shown to be associated with results favouring the interests of that sponsor. Unfortunately, sponsorship is often not disclosed in scientific articles, making it difficult to assess this potential bias. Sponsorship can consist of direct funding of the study, or of the provision of essential study materials, such as test devices. The role of the sponsor, including the degree to which that sponsor was involved in the study, varies. A sponsor could, for example, be involved in the design of the study, but also in the conduct, analysis, reporting and decision to publish. Authors are encouraged to be explicit about sources of funding as well as the sponsors role(s) in the study, as this transparency helps readers to appreciate the level of independency of the researchers |
| CHEERS | Explanation: Authors should declare anything that readers might think is a competing interest, regardless of whether the authors themselves feel their impartiality is affected. Authors may be unaware of their bias, believe they are impartial, or believe that multiple COI cancel each other out and use this to justify keeping potential COIs concealed. COI information may further help the reader interpret the credibility of the results. In the absence of a journal policy, we suggest that authors complete a standard COI form (eg, that of the ICMJE, http://www.icmje.org/conflicts-of-interest/). At a minimum, authors should declare financial interests present within 36 months before publication and any other interests that could appear to have influenced the work |
| STREGA* | There is no “explanation and elaboration” article |
| MOOSE* | There is no “explanation and elaboration” article |
| STROBE-nut | Not reported in the explanation and elaboration article (item from original STROBE) |
| TRIPOD | For models that are incorporated in guidelines, it is important to show the potential financial and other conflicts of interest of all guideline development members, not just those involved in the prediction model development |
| TRIPOD-SRMA* | There is no “explanation and elaboration” article. Suggest to check PRISMA 2020 |
| TRIPOD+AI* | There is no “explanation and elaboration” article. It recommends not use the original TRIPOD checklist |
| SPIRIT | Explanation: Competing interests, or conflicts of interest, exist when there is potential for divergence between an individual’s or institution’s private interests and their responsibilities to scientific and publishing activities. More positive outcomes, larger treatment effect sizes, and more favourable interpretation of results have been found in clinical trials with pharmaceutical industry sponsorship (Item 4) and investigators who have declared competing interests, compared to those without such interests. Although competing interests are most often associated with drug and device industries, they may exist with support from or affiliation with government agencies, charities, not for profit organisations, and professional and civic organisations. Competing interests do not in themselves imply wrongdoing. Their disclosure and regular updating enables appropriate management plans to be developed and implemented, and facilitates transparent assessment of the potential for bias. Many trials and non-industry sponsors have a conflict of interest policy for their investigators, and checklists are available to guide potential interests that should be disclosed and regularly updated by trial investigators. Types of financial ties include salary support or grants; ownership of stock or options; honorariums (eg, for advice, authorship, or public speaking); paid consultancy or service on advisory boards and medical education companies; and receipt of patents or patents pending. Non-financial competing interests include academic commitments; personal or professional relationships; and political, religious, or other affiliations with special interests or advocacy positions |
| PRISMA | Explanation: Authors of a systematic review may have relationships with organisations or entities with an interest in the review findings (for example, an author may serve as a consultant for a company manufacturing the drug or device under review). Such relationships or activities are examples of a competing interest (or conflict of interest), which can negatively affect the integrity and credibility of systematic reviews. For example, evidence suggests that systematic reviews with financial competing interests more often have conclusions favourable to the experimental intervention than systematic reviews without financial competing interests. Information about authors’ relationships or activities that readers could consider pertinent or to have influenced the review should be disclosed using the format requested by the publishing entity (such as using the International Committee of Medical Journal Editors (ICMJE) disclosure form). Authors should report how competing interests were managed for particular review processes. For example, if a review author was an author of an included study, they may have been prevented from assessing the risk of bias in the study results |
| SRQR | Explanation: Authors should identify any real or potential conflicts of interest that might have influenced or could appear to have influenced the research. Authors should also explain how these conflicts were managed in the conduct of the study, and describe the potential impact on study findings and/or conclusions. Some aspects may be mentioned as part of reflexivity (see Item 6) |
| STROBE-MR | Explanation: Financial connections between researchers and commercial or other entities and firmly held ideological or intellectual views can lead to bias in the design, conduct, or reporting of study results. When such interests are not disclosed, public trust in the research enterprise is eroded. According to the International Committee of Medical Journal Editors, “conflict of interest exists when professional judgment concerning a primary interest (such as patients’ welfare or the validity of research) may be influenced  by a secondary interest (such as financial gain). Perceptions of conflict of interest are as important as actual conflicts of interest.” Authors should erron the side of disclosing all matters that might be considered relevant by readers. |
| SQUIRE | Avoiding potential conflict of interest is as important in improvement work as it is in research. The authors in the example paper indicate the presence or absence of potential conflicts of interests, under the heading, ‘Competing Interests.’ Here, the authors provide the reader with clear and detailed information concerning any potential conflict of information. Both the original and SQUIRE 2.0 guidelines stipulate that reports of interventions to improve the safety, value or quality of healthcare should explicitly describe how potential ethical concerns were reviewed and addressed in development and implementation of the intervention. This is an essential step for ensuring the integrity of efforts to improve healthcare, and should therefore be explicitly described in published reports |
| CONSORT | Explanation: Authors should report the sources of funding for the trial, as this is important information for readers assessing a trial. Studies have showed that research sponsored by the pharmaceutical industry are more likely to produce results favouring the product made by the company sponsoring the research than studies funded by other sources. A systematic review of 30 studies on funding found that research funded by the pharmaceutical industry had four times the odds of having outcomes favouring the sponsor than research funded by other sources (odds ratio 4.05, 95% confidence interval 2.98 to 5.51). A large proportion of trial publications do not currently report sources of funding. The degree of underreporting is difficult to quantify. A survey of 370 drug trials found that 29% failed to report sources of funding. In another survey, of PubMed indexed randomised trials published in December 2000, source of funding was reported for 66% of the 519 trials. The level of involvement by a funder and their influence on the design, conduct, analysis, and reporting of a trial varies. It is therefore important that authors describe in detail the role of the funders. If the funder had no such involvement, the authors should state so. Similarly, authors should report any other sources of support, such as supply and preparation of drugs or equipment, or in the analysis of data and writing of the manuscript |
| CONSORT-abst | Explanation: Authors should report the source of funding for the trial as this is important information for readers assessing a trial. A recent systematic review showed that studies funded by the pharmaceutical industry had four times (odds ratio 4.05; 95% confidence interval 2.98–5.51) the odds of having outcomes favouring the sponsor than studies funded by other sources. Similarly, authors should report any other sources of support, such as in the preparation of the abstract, presentation, or manuscript |
| CONSORT-harms | Not reported in the explanation and elaboration article. It could be incorporated into the main CONSORT checklist |
| CONSORT-non-inferiority | Not reported in the explanation and elaboration article |
| CONSORT Cluster | Not reported in the explanation and elaboration article |
| ARRIVE | Explanation. A competing or conflict of interest is anything that interferes with (or could be perceived as interfering with) the full and objective presentation, analysis, and interpretation of the research. Competing or conflicts of interest can be financial or nonfinancial, professional or personal. They can exist in institutions, in teams, or with individuals. Potential competing interests are considered in peer review, editorial, and publication decisions; the aim is to ensure transparency, and in most cases, a declaration of a conflict of interest does not obstruct the publication or review process |
| CHERRIES* | There is no “explanation and elaboration” article |
| COREQ* | There is no “explanation and elaboration” article |
| PRISMA-P | Not reported in the explanation and elaboration article |
| TREND* | No item about COI |

*Checklists without an “explanation and elaboration” article

**Description of the variables extracted**

a) the name of the guideline (“Name of the guideline”);

b) year of publication (“Year of the publication of the guideline”);

c) type of guideline (main or extension) (“Is the guideline an extension of an existing main

guideline?” [yes/no]);

d) the name of the guideline that is extended (“Name of the extended guideline”);

e) type of extension (“Is the extension an official extension?” [yes/no/unclear]);

f) type of study addressed by the guideline (“What type of study is addressed by the

guideline?” [Clinical studies, e.g. RCT, observational studies, experimental studies//

secondary research, e.g. systematic reviews, meta-analyses // other/ not to be classified, e.g.

economic evaluations]);

g) item description and rationale (“Does the checklist provide a description for each item

and, in addition, a rationale or examples for its use?” [no description and no

rationale/examples // item descriptions provided, but no rationale/examples // both item

descriptions and rationale/examples provided]);

h) whether the guideline explicitly recommends the use of the International Committee of

Medical Journal Editors (ICMJE) disclosure of interest form (“Does the item explicitly instruct

to use the International Committee of Medical Journal Editors (ICMJE) disclosure of interest

form?” [yes/no]);

i) whether the guideline includes authorship guidance (“Does the checklist contain an item

on authorship guidance?” [yes/no]);

j) whether it provides recommendations for sharing raw data related to the study (“Does the

guideline have guidance for sharing the raw data related to the study assessed?” [yes/no]);

k) presentation of COI and sponsorship (“How is the disclosure of conflicts of interest (COI)

and sponsorship/funding presented in the guideline?” [only one item for COI is presented//

only one item for sponsorship/funding is presented// COI and sponsorship are addressed

together in a single item // two separate items for COI and sponsorship are presented// no

item for COI or sponsorship/funding // no specific item, but COI/sponsorship is mentioned

elsewhere in the text]);

l) the content of the item related to COI guidance (“Write down [copy/paste] the item in full

when the item is about COI only”);

m) the content of the item related to sponsorship guidance (“Write down [copy/paste] the

item in full when the item is about Sponsorship only”);

n) whether guidance is provided for study registration (“Is there any guidance for authors to

register their study?” [yes/no]);

o) whether the guideline recommends establishing a research protocol prior to conducting

the study (“Is there any guidance to establish a research protocol before conducting the

study? [yes/no]), and

p) whether it encourages authors to share the research protocol (“Is there any guidance

suggesting that authors share the research protocol?” [yes/no]).
